# ITS and *trnH-psbA* as Efficient DNA Barcodes to Identify Threatened Commercial Woody Angiosperms from Southern Brazilian Atlantic Rainforests

**DOI:** 10.1371/journal.pone.0143049

**Published:** 2015-12-02

**Authors:** Mônica Bolson, Eric de Camargo Smidt, Marcelo Leandro Brotto, Viviane Silva-Pereira

**Affiliations:** 1 Departamento de Botânica, Setor de Ciências Biológicas, Universidade Federal do Paraná, Curitiba, Paraná, Brazil; 2 Museu Botânico Municipal de Curitiba, Curitiba, Paraná, Brazil; Technical University in Zvolen, SLOVAKIA

## Abstract

The *Araucaria* Forests in southern Brazil are part of the Atlantic Rainforest, a key hotspot for global biodiversity. This habitat has experienced extensive losses of vegetation cover due to commercial logging and the intense use of wood resources for construction and furniture manufacturing. The absence of precise taxonomic tools for identifying *Araucaria* Forest tree species motivated us to test the ability of DNA barcoding to distinguish species exploited for wood resources and its suitability for use as an alternative testing technique for the inspection of illegal timber shipments. We tested three cpDNA regions (*matK*, *trnH-psbA*, and *rbcL*) and nrITS according to criteria determined by The Consortium for the Barcode of Life (CBOL). The efficiency of each marker and selected marker combinations were evaluated for 30 commercially valuable woody species in multiple populations, with a special focus on Lauraceae species. Inter- and intraspecific distances, species discrimination rates, and ability to recover species-specific clusters were evaluated. Among the regions and different combinations, ITS was the most efficient for identifying species based on the ‘best close match’ test; similarly, the *trnH*-*psbA* + ITS combination also demonstrated satisfactory results. When combining *trnH-psbA +* ITS, Maximum Likelihood analysis demonstrated a more resolved topology for internal branches, with 91% of species-specific clusters. DNA barcoding was found to be a practical and rapid method for identifying major threatened woody angiosperms from *Araucaria* Forests such as Lauraceae species, presenting a high confidence for recognizing members of *Ocotea*. These molecular tools can assist in screening those botanical families that are most targeted by the timber industry in southern Brazil and detecting certain species protected by Brazilian legislation and could be a useful tool for monitoring wood exploitation.

## Introduction

The Brazilian Atlantic Rainforest is one of 34 global biodiversity hotspots and is among the most seriously threatened biomes on the planet. Of the original forest, only 7 to 8% currently remains as small and biologically impoverished remnants that are exposed to strong anthropogenic pressures along the Brazilian coast [1]. Some important compilations of inventories of the floristic diversity have recorded more than 16,000 species of vascular plants in Atlantic Rainforest, with more them a half being endemic to the biome [1], List of Species of Brazilian Flora (http://floradobrasil.jbrj.gov.br/). One of its forest formations, the Mixed Ombrophilous Forest (MOF), also known as the *Araucaria* Forest, is found in the southern region of Brazil; however, well-preserved fragments of MOF occupy only 0.8% of their original range [2, 3]. Although much of this drastic reduction is associated with the expansion of agriculture and livestock, a major contribution to the intensive exploitation is the fact that MOF has been the main source of wood for furniture making and civil construction in the southern states of Brazil for more than 150 years [3, 4].

Based on lists of species published in 162 floristic and phytosociological studies, the current diversity of these forests in Paraná State is estimated to be 244 shrub-arboreal species [5]. Among the arboreal angiosperms associated with *Araucaria angustifolia* (Bertol.) Kuntze, which characterizes this forest formation, the Myrtaceae and Lauraceae families are the richest in species number, with 18 and 14 species, respectively, families that include some species complexes with problematic taxonomic delimitations and great variation regarding habits and forms [5]. In Lauraceae in particular, the precise identification of different *Ocotea* species has remained a difficult task for non-specialists, as the vegetative morphologies of these plants are very similar, their flowers are very small, and species complex delimitations are poorly defined [6, 7, 8].

The Lauraceae species from MOF, including *Ocotea porosa* (Nees & Mart.) Barroso, popularly known as “imbuia”, have historically been used in civil construction and for manufacturing furniture. Indeed, four species of *Ocotea* Aubl. and three species of *Nectandra* Rol. ex Rottb are of great interest to the timber industry and are intensely exploited and threatened [5]. The Publication of Resolution n° 278 in May 24, 2001, by the National Environmental Council (CONAMA) of Brazil, suspended the extraction or use of threatened species in the Atlantic Rainforest biome and prohibited any commercial use of a extensive list of approx. 420 native Brazilian forest species [9, 10]. Among the commercially used MOF wood species included on this list are *Ocotea catharinensis* Mez, *O*. *odorifera* (Vellozo) Rohwe, *O*. *porosa* and *Cedrella fissilis* Vel [1, 10]. Despite laws restricting the exploitation of these forest resources, clandestine logging continues almost unrestrained, and many other types of natural resources are still being exploited [9], with special focus on the internal market. Due to this global problem, since 2013, new European Union rules limit the import of illegally harvested timber and products derived from illegal timber on the EU market (http://www.euflegt.efi.int/eu-timber-regulation), including some native Brazilian species.

However, timber identification of tree species is a great challenge for monitoring and controlling the wood trade and for inspection by authorities to prevent their commercial use and illegal export. Although the most popular and routine method for identifying species is based on wood anatomy, taxonomically related species often have similar wood structure, which makes this strategy rather inefficient. Moreover, this difficulty is amplified when considering a genus or species complex with unsolved taxonomy, for which the species limits are not clearly defined. Because of their biological and economic relevance and the critical situation for the most important woody species and related taxa in MOF, these plants are ideal candidates for identification using DNA barcoding techniques. Because some taxa are protected by Brazilian Legislation [10] and listed in the Red List of Brazilian Flora [11], the feasibility of assessing DNA from dry wood could allow a broader application of DNA barcoding for wood certification or forensics tracking [12].

The molecular technique of DNA barcoding was developed as a rapid and practical way of identifying organisms at the species level using specific DNA sequences [13]. This molecular tool can be used as a forensic resource for the recognition of an unidentified organism from small amounts of processed tissue via the sequencing of a standardized genomic region. These sequences can then be compared to a set of sequences produced from previously well-identified specimens that can be easily accessed for taxonomic confirmation [14]. The molecular identification of animal species through barcoding is already well established and utilizes a portion of the gene sequence of mitochondrial cytochrome oxidase subunit I (COI) as the universal barcode standard [13]. For plants, however, the establishment of a single DNA region as a universal barcode does not appear to be a simple or realistic goal [15, 16].

Different from the situation in animals, the COI marker cannot be used in plants because of the overall lower mutation rate of plant mtDNA [17]. According to previous studies, a combination of plastid markers should be used because of the general inefficiency shown by any single marker alone [18]. In terms of biological and evolutionary features, the greatest difficulties involve the differences in evolutionary rates between taxa [19], the high frequency of interspecific hybridization events that are widespread in angiosperms [20], the incipient diversification processes in species complexes, and the poorly established interspecific limits of endemic and highly diverse plant groups [21, 22]. Finally, another factor that limits the establishment of a universal DNA barcode in plants, particularly if it is intended for applications to tree communities, is the slow evolutionary rates of perennial plants [17, 23]. Initially, the *matK* and *rbcL* regions of the plastid genome were considered promising regions for barcoding [18, 24, 25, 26]. However, low molecular resolution at the species level and problems with *matK* amplification prompted CBOL to recommend the inclusion of *trnH-psbA* [27] and, later, nrITS for different groups of angiosperms [28, 29, 30, 31].

Given the evident difficulty in determining universal regions for all terrestrial plant groups, Kress et al. [16] suggested that this molecular tool would have a greater potential if it was standardized and applied to specific or regional floristic groups. In such cases, the success of DNA barcoding could be guaranteed after determining the complementary regions (or combinations of regions) adequate for each situation. Indeed, a number of studies have demonstrated that barcodes can be satisfactorily applied to study the diversity [32] and evolution of ecological communities [33, 34], to characterize species in floristic surveys [35] and to discriminate taxa in families with complex taxonomy [36, 37, 38, 39]. Barcoding has also been useful as a forensic tool to determine the origin of commercialized natural products, such as medicinal plants [40, 41], teas [42], and foods [43]. Furthermore, the recent reports on the barcoding of commercially important species of trees [44, 45], forests communities [46, 47, 48] and CITES-protected timber species [49] have emphasized the feasibility of employing this approach as a routine method for identifying species of economic interest.

The efficiency of the barcoding approach was previously tested for Amazonian trees [48] and for Sapindaceae species from the Atlantic Rainforest [45], with both studies including species typically exploited for their wood. In these two cases, the technique appeared to contribute to the recognition of woody species, mainly those one with taxonomic confusion. Considering the economic importance of the arboreal flora of MOF and the continued illegal exploitation, barcoding the main woody tree species of these forests could be employed for controlling legal wood harvesting, detecting protected species in seized cargo, and certifying legally extracted species. Within this context, we expect this technique to be capable of correctly identifying at the species level approximately 30 important wood species distributed among 17 families. In particular, recognition of Lauraceae members at least at the genus level would be considered a great achievement because it is the most diverse family in this area and is known to be taxonomically problematic. Such an endeavor would require the following: (*i*) the establishment of a regional and multipopulational genomic DNA bank of the most important woody species in MOF; (*ii*) the determination of which DNA fragments (or combination of fragments) are useful as barcodes for this group of plants; and (*iii*) the development of a high-quality reference database of DNA barcode sequences that is easily accessible to non-academic users.

This study, which is the first to test commercially viable woody species in the Atlantic Rainforest of southern Brazil, evaluated the utility of combinations of plastidial fragments of the *matK* and *rbcL* coding regions, the intergenic spacer *trnH-psbA*, and the nuclear internal transcribed spacer (ITS) to determine (*i*) the universality of the primers and to quantify their amplification and sequencing success rates, (*ii*) their patterns of intraspecific and interspecific variation, and (*iii*) their ability to correctly identify taxa of interest.

## Materials and Methods

### Plant selection and sampling

A list of species prepared by Isernhagen et al. [5] was used to identify and select the principal timber species from MOF utilized in the commercial wood industry [50]. Based on the results, thirty arboreal angiosperms species were sampled in the field. At least three individuals from different populations were collected in Paraná State, Brazil, resulting in 112 specimens (S1 Table). This research received permission for field collection in protected areas in Paraná State, issued by Chico Mendes Institute for Biodiversity Conservation (ICMBio, Ministério do Meio Ambiente), the agency responsible for regulating activities involving natural resources in Brazil.

Samples of all specimens were deposited in the herbarium of the Botany Department of the Federal University of Paraná (UPCB), a depository of genetic patrimony, with permanent license for the storage of biological and genetic samples from Brazilian flora (59/2004—CGEN Council for the Genetic Heritage Management, Ministério do Meio Ambiente). Specialists performed identifications by comparison with herbarium samples deposited in the UPCB and Municipal Botanical Museum of Curitiba (MBM) herbaria, which contains the most important reference collection of southern vegetation in Brazil. Duplicates were sent to national (HUEFS, RB, SP) and international (NY) herbaria. Leaf tissue samples from all specimens were stored either at -80°C or in silica gel.

### DNA extraction, amplification, and sequencing

Total genomic DNA was extracted from fragments of fresh leaf tissue according to the 2X CTAB protocols [51]. Samples of total DNA were deposited in the Total DNA Bank (Laboratory of Systematic and Molecular Ecology of Plants–UFPR) and stored in ultrafreezers (-80°C).

The following fragments were used for barcodes: *matK*, with the primers *matK 3F_Kim f* and *matK 1R_Kim r* or *matK 5R* and *matK 2*.*1F* [15]; *rbcL*, with the primers *rbcLa_f* [52] and *rbcLa_r* [53]; *trnH-psbA*, with the primers *trnHf_05* [54] and *psbA3 f* [55]; and ITS, with the primers ITS92 and ITS75 [56] or ITS18_F [57] and ITS26_R [58].

Polymerase chain reactions (PCRs) were performed in 20 μL reaction mixtures containing 1 X buffer, 1.5 mM MgCl_2_, 0.2 mM each dNTP, 0.8 μM each primer, 0.4 mg/ml BSA, 0.65 unit Taq DNA polymerase, and 20–50 ng genomic DNA; 1 M betaine and 2% DMSO were added to the ITS reaction. The amplification program for each region is provided in S2 Table. The PCR products were purified with PEG 20%, and the sequencing reactions were performed by Macrogen Inc. (Seoul, South Korea—http://dna.macrogen.com) using the same primers as used for PCR (S2 Table).

### Barcoding analyses

The forward and reverse sequences were edited, and consensus sequences were obtained using the Staden Package software [59]. Multiple sequence alignments were performed using Clustal W program [60] and MEGA6 software [61] with the standard parameters, and the matrices were confirmed manually. To facilitate the alignment of the matrices for the two spacers (*trnH-psbA* and ITS), the sequences were organized according to the taxa classification by APG III [62]. All of the sequences generated in this work were submitted to International Nucleotide Sequence Database Collaboration GenBank (Accessions, *rbcL*: KF561901—KF561971; *matK*: KF555384—KF555448; ITS: KF420934—KF421013; *trnH- psbA*: KF421014—KF421108 (S2 Table)).

#### Distance-based methods

The genetic variability of each marker was described by the median length (bp) and total alignment length (bp); the number of polymorphic sites and number of parsimony informative sites (PIC), both discounting gaps; the number of sites with gaps; nucleotide diversity (Pi); and the number of haplotypes in each region using DnaSP 5.10 [63].

The barcoding analyses were conducted separately for each region and combined in the following arrangement: (*i*) coding regions (*matK* + *rbcL*); (*ii*) non-coding regions (*trnH-psbA* + ITS); (*iii*) plastid genome regions (*matK* + *rbcL* + *trnH-psbA*); and (*iv*) the four regions combined (*matK* + *rbcL* + *trnH-psbA* + ITS). In the two latter combinations, only species that had sequences from at least two and three regions, respectively, were considered.

The presence of ‘barcoding gaps’ between the interspecific and intraspecific distances were evaluated using frequency histograms based on the uncorrected paired p-distances obtained using the taxondna program and the ‘best close match’ test [64].

The ‘best close match’ test was used to determine the closest correspondence of a target sequence with all of the others in a set of aligned data, there by establishing a similarity limit based on the distribution of the frequencies of the intraspecific and interspecific distances. A limiting value was calculated separately for each matrix (all makers separately and in combination), and the relative frequencies of all of the intraspecific distances were calculated to determine a limiting value below 95% [64]. In such tests, sequences can be classified as follows: “correct”, when the genetic distances between the target sequence and other sequences of the same species fit within 95% of the calculated limit; “ambiguous”, when a sequence lies within this percentage but is similar to that of another species; “incorrect”, when a target sequence does not encounter a sequence of the same species within the calculated limit; and “incongruent”, when the sequence does not lie within the 95% range of the calculated limit [65].

#### Monophyly-based methods

The following different phylogenetic approaches were used to evaluate whether the samples from different individuals form a species-specific clusters: distance methods applying a paired p-distance with the Neighbor-joining algorithm (NJ) [66, 67]; Maximum Likelihood (ML) methods using an evolutionary model selected with Bayesian Information Criterion [61]; and Maximum Parsimony (MP), which uses heuristic searches with 2000 replicates and random additions retaining 20 trees per replication with TBR algorithm, followed by an analysis of TBR to explore all of the topologies obtained in the initial analysis, establishing a limit of 10,000 trees. A strict consensus tree was constructed from multiple equally parsimonious trees. Support analyses utilized 1000 bootstrap replicates (BP) [68] in the NJ and MP analyses. For computational reasons, only 100 bootstrap replicates were used for ML. The NJ and ML analyses were performed using MEGA6 software [61], and the MP analyses were performed using PAUP v.4.0b10a software [69] with Fitch parsimony [70]. The phylogenetic trees were rooted in Lauraceae, basal angiosperm family, in all of the analyses.

## Results

### Amplification, sequencing success and makers features

A genomic DNA collection was assembled using 112 individuals from 30 different timber angiosperms species of high commercial value in the MOF. PCR was performed for all 112 accessions using ITS, 108 using *trnH-psbA*, 102 using *matK* and a lower sampling of 70 using *rbcL*. The amplification reactions were performed with high success (>90%) for *trnH-psbA*, *rbcL*, and ITS, whereas *matK* demonstrated lower reaction efficiency, with successful amplification of only 74% of the samples (Table 1). Multiple attempts per sample were required for *matK* for adjustment of the DNA template concentration and identifying ideal primer pairs, which demonstrated significant variability in amplification success among the different botanical families.

**Table 1:** Sample sizes, success rates of amplification and sequencing, and the molecular characteristics of the four markers evaluated for woody species from MOF.

Barcode	*N* of samples tested	*N* of samples amplified (%)	*N* of samples sequenced (%)	Median length (bp)	Alignment length (bp)	Number of polymorphic sites*	Number of parsimony informative sites (PIC)*	Number of sites with gaps	Nucleotide diversity (Pi)	*N* accessions (*N* species)	Number of haplotypes
ITS	112	103(91)	80(78)	656.4	850	179	156	544	0,207	80(29)	44
*trnH-psbA*	108	106(98)	89(84)	482.2	707	101	96	584	0,31	89(30)	44
*matK*	102	75(74)	65(86)	817.5	939	438	376	255	0,168	65(28)	52
*rbcL*	70	68(97)	68(100)	571.8	580	156	144	17	0,069	68(30)	33

*excluding those with gaps/missing data.

High-quality sequences were obtained for most of the amplified DNA samples. *rbcL* exhibited the highest efficiency, with 100% sequencing success and *matK* (86%) and *trnH-psbA* (84%) intermediate efficiency; in contrast, ITS showed the least efficiency, with 78% success (Table 1). The four DNA regions chosen were effectively amplified and sequenced for most of the species, generating a database of 302 sequences; however, it was not possible to obtain sequences for all of the DNA regions for all accessions (S1 Table). When we discarded the accessions that were not amplified and/or sequenced for two or more fragments, analyses were performed with a final matrix composed of 80 sequences of ITS, 89 of *trnH-psbA*, 65 of *matK* and 68 of *rbcL*, from 101 accessions (Table 1).

Based on multiple alignments of all sequences for each region, *matK* presented the largest length (939 bp) and *rbcL* the shortest length (580 bp) (Table 1). The number of polymorphic sites, discounting gaps, varied for individual regions: from 101 in *trnH-psbA* to 438 in *matK*. The largest number of parsimony informative characters was provided by *matK* (376), followed by ITS (156) and *rbcL* (144). *trnH-psbA* showed the highest number of gaps (584) and the highest nucleotide diversity (0.31), followed by ITS with 544 gaps and nucleotide diversity of 0.207. The greatest number of haplotypes was generated by *matK* (52), followed by ITS and *trnH-psbA* (44 each) (Table 1). The haplotypes provided by these three markers were species-specific, except for some Lauraceae species. *rbcL* and *trnH-psbA* provided only one haplotype for all *Nectandra* species and *matK* provided two, but one shared between the three species (*N*. *grandiflora*, *N*. *lanceolata*, *N*. *megapotamica*). For *Ocotea rbcL* provided four, *trnH-psbA* seven and *matK* eight haplotypes, being one or more haplotypes shared between four from five species analyzed, including those most important taxa (*O*. *porosa*, *O*. *puberula*, *O*. *odorifera* and *O*. *catharinensis*). ITS provided the most specific haplotypes, with only one haplotype shared between all accessions of *Nectandra megapotamica* and *N*. *lanceolata*. All other species were characterized by one (*O*. *catharinensis*, *O*. *odorifera*, *O*. *puchella*) to three exclusive ITS haplotypes (*O*. *porosa*).

### Comparative performance of barcodes

#### Testing discrimination of species based on distance methods

**Fig 1:**
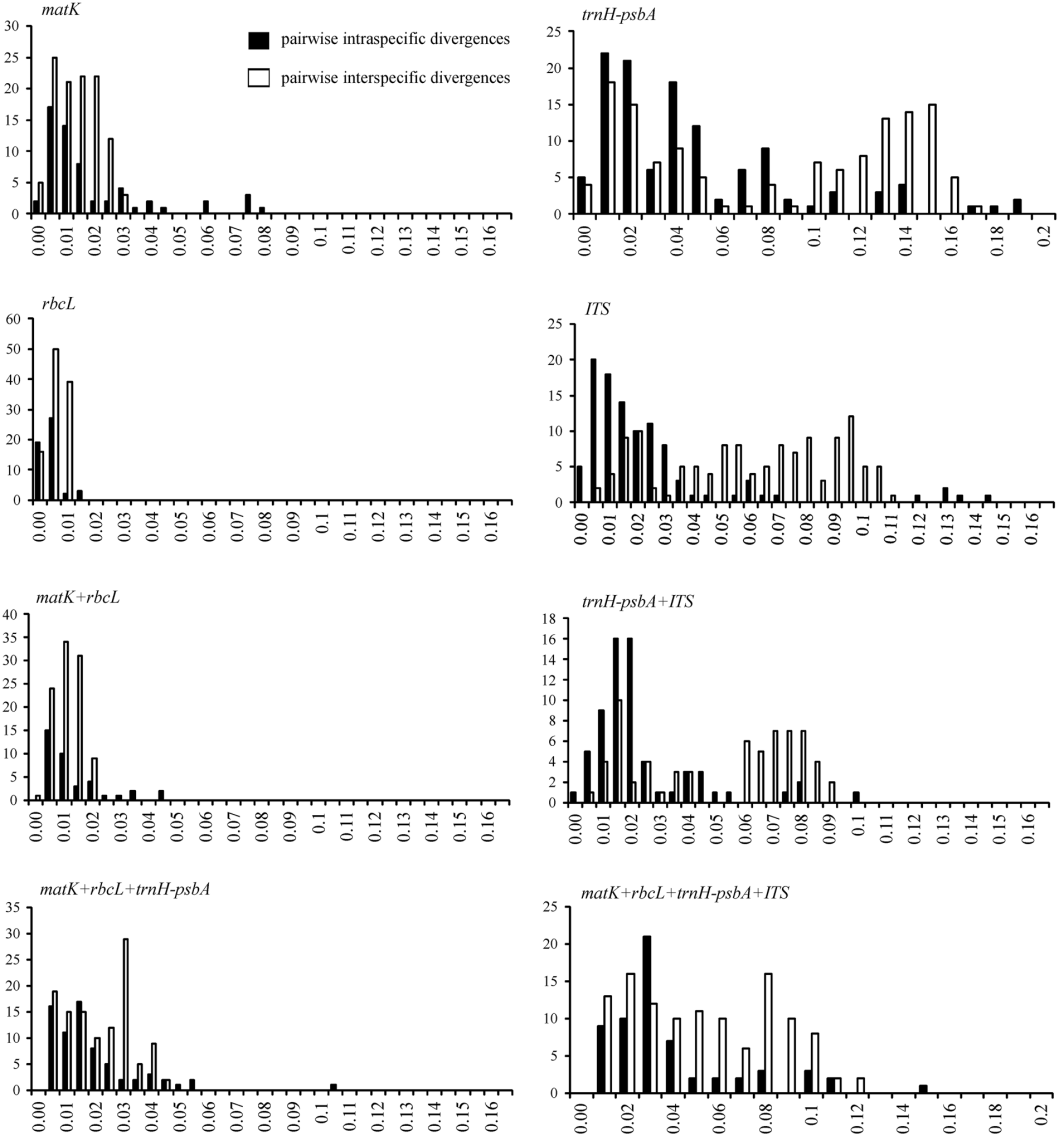
Histograms of the frequencies (*y*-axes) of pairwise intraspecific (black bars) and interspecific (white bars) divergences based on the p-distance (*x*-axes) for individual and combined ITS, *trnH-psbA*, *matK* and *rbcL* markers.

As estimated from p-distances, variations in intra- and interspecific distances were observed among the four DNA regions. The presence or absence of a barcoding gap for individual or combined regions was represented by the frequency distribution of the genetic distance between intraspecific sequences and between interspecific sequences (Fig 1). When loci were individually analyzed, ITS presented the best barcode gap performance, with 71% of pairwise interspecific p-distances greater than 0.05 and 85.7% of pairwise intraspecific p-distances lower than 0.05. Although *trnH-psbA* provided the highest values of interspecific genetic distances, with a range varying from zero to a maximum of 0.19, this was combined with a high frequency of low distances at the interspecific level. Nonetheless, complete discontinuity was not found between the intra- and interspecific ranges for these two individual markers. The best barcode gap performance appears to have resulted from the combination of ITS + *trnH-psbA*. Conversely, *rbcL* showed the worst performance, with a total overlap of intra- and interspecific variation; the same unsatisfactory result was observed for *matK* and for the combination *matK + rbcL* (Fig 1).

**Fig 2:**
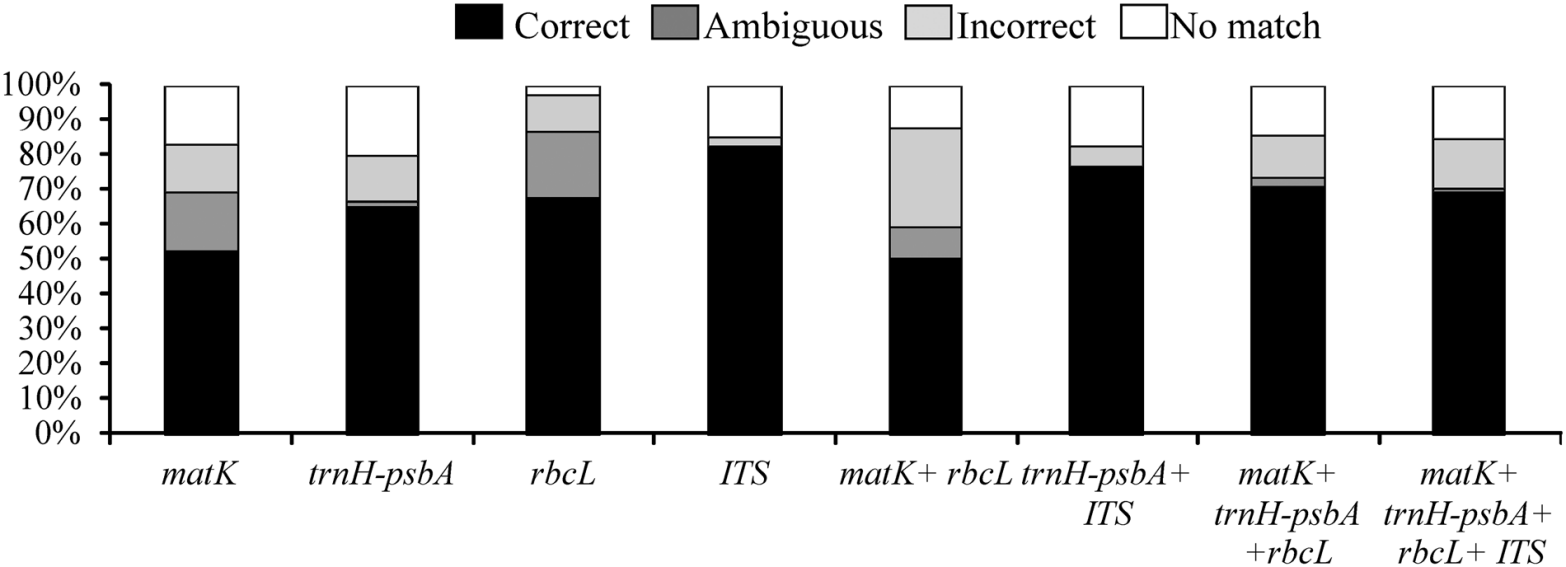
Success of species identification based on individual and combined analyses of ITS, *trnH-psbA*, *matK* and *rbcL* markers using the taxondna program and the ‘best close match’ method.

The results of the ‘best close match’ test for individual regions revealed ITS as the best at species identification (83%), followed by *rbcL* (68%), *trnH-psbA* (65%) and *matK* (52%). The combined regions showed correct identification of nearly 76% for *trnH-psbA +* ITS, 71% for *matK + trnH-psbA + rbcL*, and 69% for *matK + trnH-psbA + rbcL +* ITS (Fig 2). In contrast, the *matK + rbcL* combination exhibited the worst performance for correct identification and the largest proportion of incorrect identifications. Ambiguous identification rates were relatively low (< 9%) for most of the individual regions and combinations.

#### Testing discrimination of species based on phylogenetic trees

Determination of the percentages of species-specific clusters was based on the reconstruction of phylogenetic trees according to three analyses (NJ, ML, and MP) for the four regions and four combinations tested. The phylogenetic trees generated from the three analyses differed in terms of their topologies and degrees of resolution, though there was little difference between the percentages of species-specific clusters obtained with ML and MP. The trees generated using NJ did not have good resolution and only demonstrated well-resolved topology for the ITS region and the combination *trnH-psbA +* ITS (77% support). The low resolution and low support values for the NJ trees prompted us to concentrate our efforts on ML and MP analyses (Table 2; S3 Table).

**Table 2:** Percentage of species-specific clusters using different tree-based methods (NJ, MP, and ML) with ≥70% bootstrap support for single DNA regions and different combinations.

DNA region	*N* species	NJ	ML	MP
*matK*	20	50	50	45
*trnH-psbA*	25	56	64	72
*rbcL*	25	68	72	68
ITS	22	77	91	96
*matK + rbcL*	20	65	65	65
*trnH-psbA +* ITS	22	77	91	86
*matK + trnH-psbA + rbcL*	25	64	76	76
*matK + trnH-psbA + rbcL +* ITS	24	62.5	75	75

**Fig 3:**
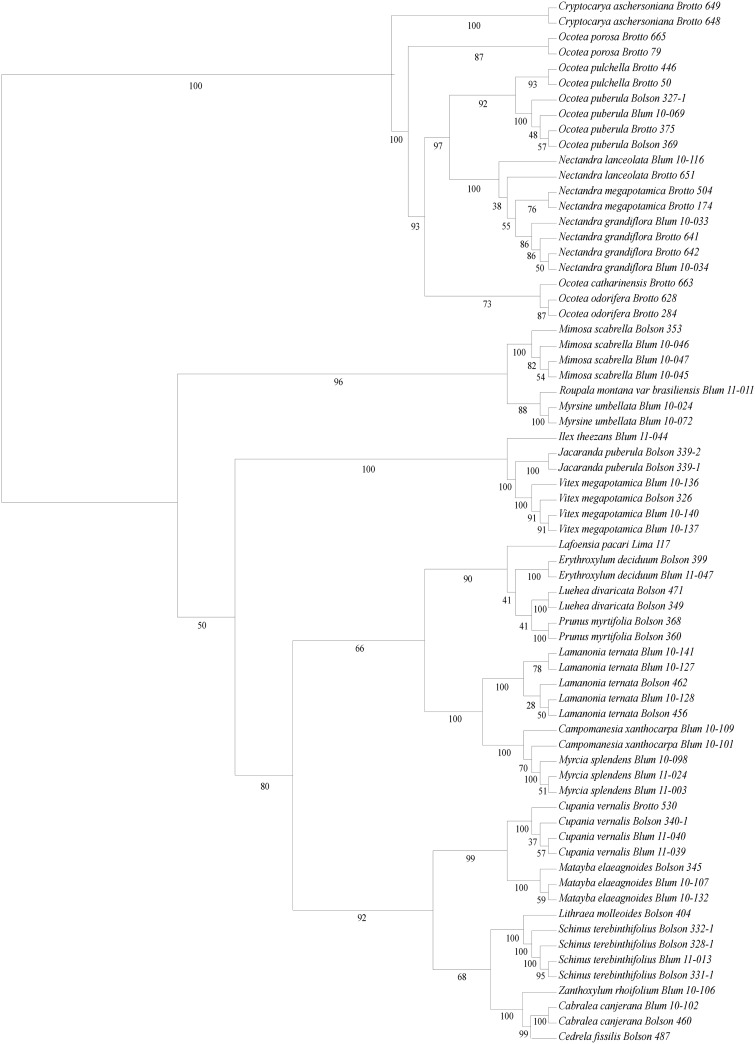
Cladogram for *trnH-psbA +* ITS generated by Maximum Likelihood. The bootstrap values ≥70% are shown under the branches. The species name is followed by the accession number of the specimen.

The topology generated by MP demonstrated the greatest support values for the ITS region, which was capable of recovering 96% of species-specific clusters with ≥70% of bootstrap support, followed by *trnH-psbA* (72%) and *rbcL* (68%). Conversely, ML revealed a greater resolution for *rbcL* (72%). As the worst performance, *matK* demonstrated the same percentages with both ML and NJ (50%) (Table 2). When compared with MP analysis, ML showed a more resolved topology for internal branches in the combined regions, *trnH-psbA +* ITS, with 91% of species-specific clusters and ≥70% of bootstrap support (Fig 3). Therefore, we choose to explore in deeper detail the *trnH-psbA +* ITS tree generated by ML; we present the ITS tree obtained by MP in the supporting information section (S1 Fig).

The *trnH-psbA* + ITS tree inferred by ML discriminated 100% of 16 sampled families (Fig 3). Among them, there was only one specimen analyzed for Rutaceae (*Zanthoxylum*), Lythraceae (*Lafoensia*), Aquifoliaceae (*Ilex*) and Proteaceae (*Roupala*). With more than one sample per species, families Cunoniaceae (*Lamanonia*), Rosaceae (*Prunus*), Malvaceae (*Luehea*), Erythroxylaceae (*Erythroxylum*), Lamiaceae (*Vitex*), Bignoniaceae (*Jacaranda*), Myrsynaceae (*Myrsine*) and Fabaceae (*Mimosa*) were discriminated with high support (100% BP). Families with more than one species in different genera, such as Meliaceae (*Cedrela* and *Cabralea*), Anacardiaceae (*Lithraea* and *Schinus*), Sapindaceae (*Cupania* and *Matayba*), Myrtaceae (*Myrcia* and *Campomanesia*), were also discriminated with high support (99–100% BP). Only Lauraceae, with three genera (*Cryptocarya*, *Ocotea* and *Nectandra*) and nine species sampled, was not completely discriminated. The genus *Cryptocarya* was distinguished as a sister group of *Ocotea* and *Nectandra*, with 100% bootstrap, yet species of *Nectandra*, which were also identified with 100% support, were included within the genus *Ocotea*. *Nectandra* presented an intermediate degree of discrimination (76–85% BP) between *N*. *megapotamica* and *N*. *grandiflora*, though samples of *N*. *lanceolata* were not grouped together and appeared as successive sister groups of the other species in the genus. Of the five sampled *Ocotea* species, *O*. *porosa* was discriminated with good support (87% BP), followed by *O*. *catharinensis* and *O*. *odorifera* with moderate support (73–87% BP). The multiple individuals of *Ocotea pulchella* (93% BP) and *O*. *puberula* (100% BP) were grouped as a highly supported species-specific clade (97% BP) together with *Nectandra* species, making *Ocotea* paraphyletic in our analyses.

## Discussion

This is the first report of the efficacy of four barcode loci in identifying commercial wood tree species from MOF in the southern Brazilian Atlantic Rainforest. Combining the characteristics of molecular variation, intra- and interspecific divergence patterns, the ‘best close match’ test and the ability to recover species-specific clusters in phylogenetic trees, our results support the designation of ITS as the principal barcode locus for the forensic identification of the main explored timber species in MOF. The inclusion of *trnH-psbA* as an additional locus is recommended because, together with ITS, the barcode combines nuclear and plastidial genomic information and confers more reliability to the data set though not increasing the power of taxonomic identification of species.

### Amplification, sequencing success and makers features

According to the CBOL plant working group, an ideal DNA barcode must combine conserved regions for universal primer design, show high rates of PCR amplification and sequencing, and present genetic variability that is high enough to distinguish sequences at the species level yet sufficiently conserved among individuals of the same species [13, 14, 18]. The first step in determining whether a DNA fragment can serve as a barcode is to evaluate its universal applicability by quantifying PCR and sequencing success. Indeed, the ease of obtaining a high-quality sequence determines whether DNA barcoding can become a viable technique for monitoring woody cargo and product inspection by regulatory agencies. In this regard, the *rbcL* plastid DNA region produced more successful reactions and specific amplification and resulted in simple and high-quality sequencing for MOF species, showing the best performance, with 100% of the samples successfully sequenced. Although previous studies have described the use of one or two primer pairs to sequence ≥90% of samples [28, 35], only one was necessary to amplify all of our accessions.

In our study, *trnH-psbA* was easily amplified from 98% of the samples and showed intermediate success in sequencing (84%). A similar pattern has been reported for other land plant groups, with *trnH-psbA* PCR amplification and sequencing rates high enough to be considered as barcode loci [16, 47]. Although this marker has been rejected as barcodes in some cases due to the length (> 1000 bp) and difficulty in bidirectional sequencing [18, 21], we were able to obtain satisfactory success rates of PCR amplification and sequencing as well as high-quality bidirectional sequences.

Among the four fragments, ITS presented a high success rate for amplification (91%) but the worst performance for sequencing (78%). Similar results were found in other studies in which the authors did not report difficulty in amplifying ITS but did encounter problems in sequencing this locus in Cycadales species and other gymnosperms [36], in tree species of India [47], and in tropical Amazonian trees [48]. This common pattern of ITS has been linked to secondary structure formation, multiple copies from paralogs, endophytic fungal contamination or wide variations in sequence lengths, leading to low-quality sequences [30]. In fact, this drawback has been used as an argument for considering this nuclear spacer as less acceptable as a standard DNA barcode in some research (e.g., [38]). Despite showing sequencing problems in some studies, this region has been suggested to be a good complement to core barcode fragments, particularly in cases in which the laboratory expense for producing sequences was not so costly or the very high species-discriminating rate compensated for the investment [31, 45, 47, 48]. Although the major problem related to ITS is the occurrence of divergent paralogs, resulting in nonspecific amplifications that are often identified by the presence of multiple bands on an agarose gel [30], this issue was not observed for the woody species from MOF. Considering that we did not encounter indications of this and the aforementioned problems associated with ITS, the low rate of sequencing is the only inconvenience that needs to be addressed.

Although *matK* exhibited the lowest amplification performance, this locus generated an intermediate sequencing percentage (86%). Neither of the two most frequently used *matK* primer pairs was efficient for *Erythroxylum deciduum* A. St.-Hil. (Erythroxylaceae) and *Vitex megapotamica* (Spreng.) Moldenke (Lamiaceae) DNA amplification. Many studies indicate that *matK* is a key marker for discriminating specific groups [71,72]; nevertheless, many authors have questioned its utility as a barcode due to poor amplification and sequencing performance and problems related to the universality of the primers [22, 36, 37, 39, 73, 74]. Based on the accumulated knowledge in the literature as well as our own experience, to obtain good results with *matK*, it is necessary to utilize different published primers according to the plant family or to propose new primers for the samples of interest [75, 76]. For MOF woody species, multiple attempts per sample were required to adjust the concentrations of DNA template and to identify the ideal primers pairs for *matK*, a marker that demonstrated significant variability in amplification success among different families. Because of these characteristics, *matK* appears to be an impractical choice as a forensic barcode for plants that are phylogenetically unrelated, as is the case for the woody species from MOF.

In terms of molecular variation, *rbcL* was the most conserved sequence among the four regions, as indicated by the slight difference between the median sequence length and alignment length and the very low number of gaps in the final matrix. Although the *trnH-psbA* intergenic spacer demonstrated the lowest number of polymorphic sites, this locus presented the highest number of gaps due to the great variation in sequence length. As reported in many studies [18, 21, 22, 24, 77], this molecular pattern makes it somewhat difficult to align a matrix containing a large number of distinct taxonomic families, and this region has been not recommended for barcoding in those cases. Accordingly, we opted to carry out the alignment by grouping sequences according to orders and families following the APG III classification [62], resulting in a more reliable matrix.

When we compare the amplification and sequencing success of different DNA regions is observed that it is relatively harder to obtain sequences of ITS and *matK*; however, as discussed below, ITS was the best marker in identifying species from MOF. In this way, the low sequencing rate of ITS is a difficulty that we must deal with.

### Comparative performance of markers

#### Testing discrimination of species based on distance methods

The combination of the *rbcL* and *matK* regions has been recommended as a universal barcode for land plants [18, 53] and when compared to individual regions, has been shown by a number of studies to result in better resolution for discriminating between species [22, 38, 73]. However, this combination was able to correctly identify only 50% of the commercially important woody species from the MOF, demonstrating less accuracy than the individual regions (*matK*, 52%; *rbcL*, 68%) and indicating it as an inappropriate barcode gap. This combination also demonstrated similar low efficiency (50%) in discriminating species of *Primula* L. [73] and Labiatae [39] within a strict taxonomic context, making it difficult to justify the use of core barcodes to distinguish between these closely related species. In a floristic approach, however, these markers were able to discriminate approximately 70% of a large list of different families from the flora of Wales [35]. Despite the low variation, *rbcL* was more efficient in distinguishing among woody MOF species using taxondna when compared to *matK*, which showed a high number of variable sites and the worst performance in recognizing taxa. This result was unexpected because *matK* is known to be a rapidly evolving coding region, and this marker has been used as a barcode with an impressive ability to discriminate among angiosperm species [18, 24, 39, 71, 74]. Regardless, our results could be explained by a technical artifact due to the low amplification/sequencing success of *matK*, resulting in a smaller number of analyzed taxa, combined with the effect of sampling bias when studying a non-related set of families, which could be better recognized by a slower evolving marker such as *rbcL*.

ITS exhibited the best species discrimination performance according to the ‘best close match’ test using taxondna; this single fragment was more efficient in identifying species than any other region or combination for the woody species studied. As has been observed in other studies, ITS provided a good alternative for the molecular identification of phylogenetically unrelated species and, at the same time, provided accurate identification of closely related species [31, 40, 41, 45, 78]. Although *trnH*-*psbA* did not show great performance alone, in combination with ITS, it was capable of providing accurate identification for more than 75% of woody MOF species.

When all possible combinations were evaluated for all fragments explored in the woody species from MOF, we determined that the best combinations were *trnH-psbA +* ITS and *matK + trnH-psbA + rbcL*. These combinations did not generate ambiguous identifications, with only low levels of incorrect identification, recognizing >76% and >71% of the species, respectively. Roy et al. [22] similarly reported good results with *trnH-psbA +* ITS for identifying species of Indian *Berberis* L and in a large-scale biodiversity inventory of tropical tree species of India [47]. In spite of the significant sequencing investment required for the four regions, we conclude that none of the combinations was more efficient than ITS alone for the MOF woody species examined in this study.

#### Testing discrimination of species based on phylogenetic trees

One of the most efficient ways to determine the utility of a DNA region as barcode is to verify whether it can detect species-specific clusters of species from the same genus and family using a phylogenetic analysis. In general, commercially important woody species of different genera are grouped within their respective families; this also occurred with species for which only a single individual was analyzed. Most species with multiple individuals form species-specific clusters, including those of Lauraceae, with a high confidence to identify the most threatened *Ocotea* species.

In this regard, MP analysis produced cladograms with better resolution for ITS and with the greatest percentage of species-specific clusters, considering all clusters with more than 70% of bootstrap. Using the same criterion of support, the four different combinations tested presented better results by ML analysis. *trnH-psbA +* ITS and *matK + trnH-psbA + rbcL* demonstrated the best topology, with 91% and 76% of the species being grouped into species-specific clusters, respectively. Moreover, all of the cladograms from the three analyses of the combination *trnH-psbA +* ITS produced only species-specific clusters.

Recent reports of barcoding commercially important species of trees [44], forest communities [46, 47] and CITES-protected timber species [49] have been illuminated the feasibility of using this approach as a routine method for identifying species of economic interest. As an example, in studies carried out with Meliaceae, ITS provided the best resolution for interpreting species-specific clades in *Cedrela*, a broad genus known for its importance as a wood supply [49]. In addition to Lauraceae and Meliaceae, Sapindaceae from the Atlantic Rainforest [45] and Amazonian trees [48] are species of great economic importance, and many taxonomic challenges need to be solved. In these cases, barcoding techniques appear to contribute to the recognition of key woody species, mainly those with some taxonomic confusion. For species threatened by illegal exploitation, the use of ITS as a barcode affords the possibility of adding an auxiliary tool for monitoring the use of their wood worldwide [45, 48, 49].

In our study, the ITS region and the combination *trnH-psbA* + ITS efficiently distinguished with high support three genera of Lauraceae (*Cryptocarya*, *Ocotea* and *Nectandra*) from woody species from MOF. Even for closely related species of *Ocotea*, the ITS region and the combination *trnH-psbA* + ITS presented similar potential for distinguishing among species by detecting species-specific clusters for all taxa. In this way, our results also indicated barcoding as a promising tool to molecularly recognize the main woody *Ocotea* species that are actually explored and threatened using the fragments studied to date. The same DNA regions were recently tested in 42 species from 11 genera of Lauraceae, and the results showed that *trnH-psbA* could efficiently identify approximately 82% of the species [79], except for some *Ocotea* such as *O*. *odorifera* and *O*. *puberula* (Rich.) Nees, the only two commercially viable woody species from MOF examined.

Some studies have stressed the non-monophyly origin of some Lauraceae genera, a fact that is responsible for the lack of resolution with regard to the systematics of this family [6, 7]. A proper phylogenetic study on Brazilian representatives of this family would require increased sampling and the inclusion of other genera, such as *Caryodaphnopsis*, *Clinostemon*, *Dicypellium* and *Mezilaurus*, which are not native to MOF or are not explored as sources of wood. For *Ocotea* in particular, which is composed of some species complexes with poorly defined limits among taxa, more works focusing on morphological and genetic variation at population level are required to address the relationship between closely related species. In this way, DNA barcoding could play an important role in the molecular recognition of important species in a way that is complementary to traditional methods of identifying timber, with no claim of resolving taxonomic and evolutionary questions. The feasibility of using dry tissue fragments for DNA extraction and genetic analysis has a major impact on barcoding as a forensic tool in the regulation of endangered tree species trade [12], including some Lauraceae species listed in the Red List of Brazilian Flora [11], or wood Eco-certification for product manufacturing.

Given the difficulty of classic taxonomic identification of very close *Ocotea* and *Nectandra* species found in MOF, it is expected that other species also suffer from collection pressure, even though they are not as useful to industry. Although only *Ocotea porosa*, *O*. *catharinensis* and *O*. *odorifera* are on the list of taxa banned for exploration, other *Ocotea* species could also be partially impacted, which may indicate targeted extraction. In this case, we cannot assert that the observed excellent resolution of certain barcodes for detecting species-specific clusters will be maintained if the species list is extended to all *Ocotea* or *Nectandra* species found in southern Brazil. Nonetheless, although this tool may not accurately identify all species, recognizing a genus is a major breakthrough for establishing rapid methods for screening seized cargo and the supervision of manufactured products. In cases in which a specific identification of another species of Lauraceae not represented here is essential, we recommend the use of other highly variable regions that have undergone rapid evolution [27] in association with established barcode regions, tiered barcoding system [80] and/or studies at population levels. According to Feng et al. [80] a tiered barcoding system was an efficient tool for *Populus*, which is an ecologically and economically important genus of trees, but distinguishing between wild species is relatively difficult due to extensive interspecific hybridization and introgression, and the high level of intraspecific morphological variation.

### Conclusion

One of the main objectives for establishing these molecular barcodes is to provide a practical forensic tool to determine the legality of the raw materials extracted from protected forests [34, 46, 47, 48] or from specific botanical families of interest to the timber industry [44, 45, 49]. The use of the ITS region could increase the laboratory time required for editing sequences or repeating sequencing reactions. Regardless, based on the practicality and viability of barcoding as a routine technique, we recommend the ITS region for barcode identification of these species, as it demonstrated a high degree of identification success using taxondna and a better resolution of species-specific clusters in the phylogenetic tree generated by Maximum Parsimony, a computational method currently used in practice. Although exercising caution with regard to affirming this technique as the ultimate solution for identifying wood resources from MOF, our results show a great potential for recognizing those families most frequently explored, with high support at the genus level and relative confidence at the species level. This could be the first step in providing instruments for Brazilian authorities to follow the example of the new European Union rules for the timber trade, which since 2013, prohibits operators in Europe from placing illegally harvested timber and products derived from illegal timber on the EU market. Considering the great diversity of tree species in tropical forests, these molecular tools can assist in screening those botanical families most targeted by the timber industry and in detecting those species protected by Brazilian legislation.

## Supporting Information

S1 Fig Cladograms for ITS generated by Maximum Parsimony. The bootstrap values ≥70% are shown under the branches. The species name is followed by the accession number of the specimen.
(TIF)

S1 Table Collection details for the commercial woody specimens used from MOF and accession numbers to International Nucleotide Sequence Database Collaboration GenBank. Plant classification follows APG III (2009);—indicates no sequence obtained.
(DOCX)

S2 Table Primers and PCR conditions for plastid and nuclear DNA sequence amplifications in this study. The complete references can be found in the text.
(DOCX)

S3 Table Percentage of species-specific clusters (SSC) and non-species-specific clusters (NSSC) using different tree-based methods (NJ, MP, and ML) with different bootstrap support for single DNA regions and different combinations. (DOCX)
