# Supplementary figures and images for: ITS and trnH-psbA as Efficient DNA Barcodes to Identify Threatened Commercial Woody Angiosperms from Southern Brazilian Atlantic Rainforests
Source: PLoS One. 2015 Dec 2;10(12):e0143049. doi: 10.1371/journal.pone.0143049 (PMC4704546; doi:10.1371/journal.pone.0143049)

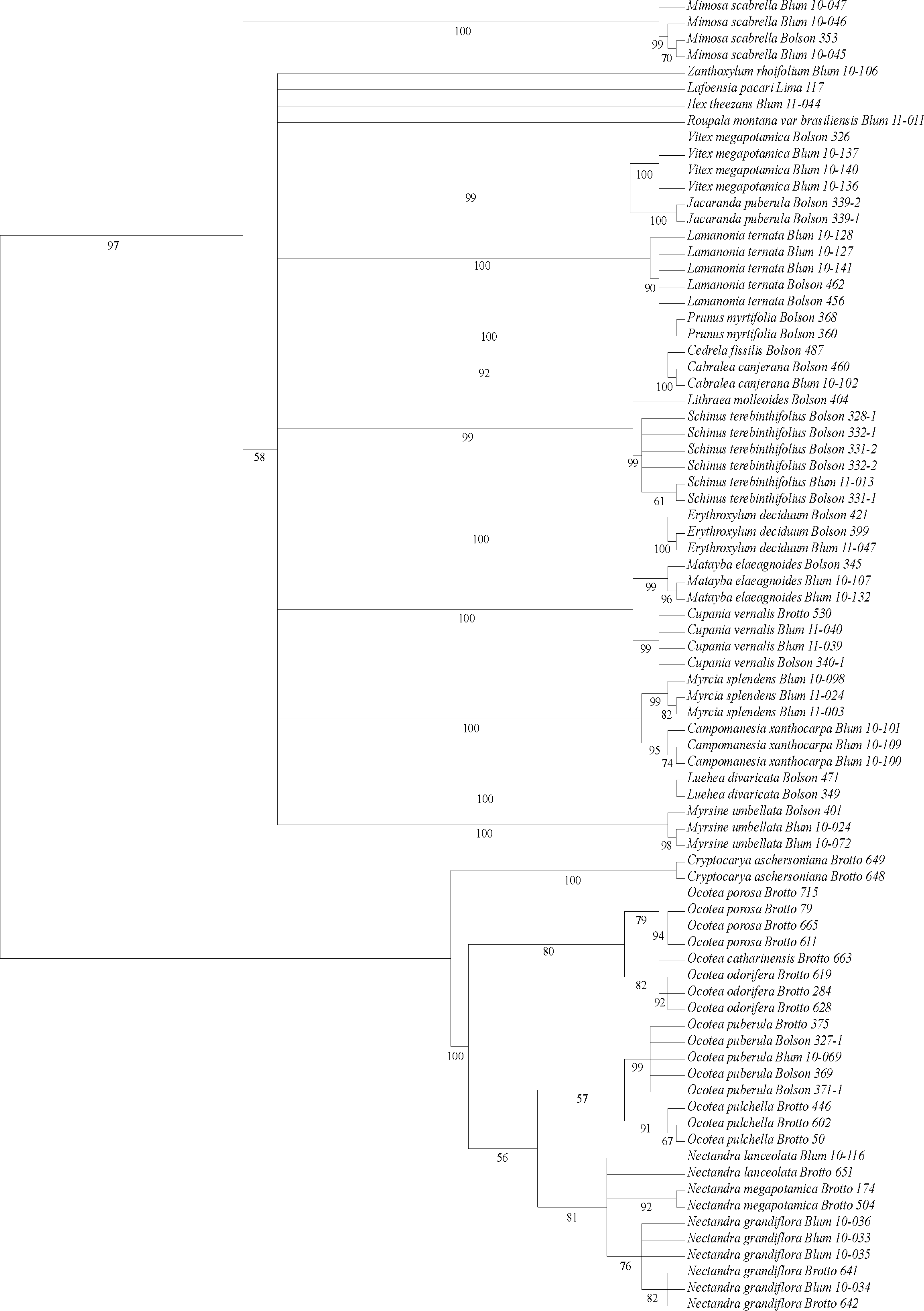

Supplement: S1 Fig — The bootstrap values ≥70% are shown under the branches. The species name is followed by the accession number of the specimen. (TIF) [file pone.0143049.s001.TIF]
