# Supplementary material for: ITS and trnH-psbA as Efficient DNA Barcodes to Identify Threatened Commercial Woody Angiosperms from Southern Brazilian Atlantic Rainforests
Source: PLoS One. 2015 Dec 2;10(12):e0143049. doi: 10.1371/journal.pone.0143049 (PMC4704546; doi:10.1371/journal.pone.0143049)
Supplement: S1 Table — Plant classification follows APG III (2009);—indicates no sequence obtained. (DOCX) [file pone.0143049.s002.docx]

**S1 Table. Collection details for the commercial woody specimens used from MOF and accession numbers** **to International Nucleotide Sequence Database Collaboration GenBank.** Plant classification follows APG III (2009); - indicates no sequence obtained.

| **Order/ Family/ Species** | **Voucher specimen** | **Location (city)** | **GenBank access number** | | | |
| --- | --- | --- | --- | --- | --- | --- |
|  |  |  | ***matK*** | ***trnH-psbA*** | ***rbcL*** | **ITS** |
| LAURALES |  |  |  |  |  |  |
| **Lauraceae** |  |  |  |  |  |  |
| *Cryptocarya aschersoniana* Mez | Brotto ML 649 | Telêmaco Borba | KF555389 | KF421020 | KF561909 | KF421006 |
| *Cryptocarya aschersoniana* Mez | Brotto ML 648 | Telêmaco Borba |  | KF421021 | KF561908 | KF421007 |
| *Nectandra grandiflora* Nees & C. Mart. ex Nees | Blum CT 10-033 | Tijucas do Sul | KF555420 | KF421022 | KF561939 | KF420973 |
| *Nectandra grandiflora* Nees & C. Mart. ex Nees | Brotto ML 641 | Telêmaco Borba | KF555421 | KF421024 | KF561940 | KF420969 |
| *Nectandra grandiflora* Nees & C. Mart. ex Nees | Brotto ML 642 | Tijucas do Sul |  | KF421025 | KF561941 | KF420968 |
| *Nectandra grandiflora* Nees & C. Mart. ex Nees | Blum CT 10-034 | Tijucas do Sul |  | KF421023 |  | KF420972 |
| *Nectandra grandiflora* Nees & C. Mart. ex Nees | Blum CT 10-035 | Tijucas do Sul |  |  |  | KF420971 |
| *Nectandra grandiflora* Nees & C. Mart. ex Nees | Blum CT 10-036 | Tijucas do Sul |  |  |  | KF420970 |
| *Nectandra lanceolata* Nees | Blum CT 10-116 | Colombo | KF555422 | KF421026 | KF561942 | KF420967 |
| *Nectandra lanceolata* Nees | Brotto ML 651 | Mato Rico | KF555424 | KF421028 | KF561943 | KF420966 |
| *Nectandra lanceolata* Nees | Blum CT 10-117 | Colombo | KF555423 | KF421027 | KF561944 |  |
| *Nectandra megapotamica* (Spreng.) Mez | Brotto ML 174 | São José dos Pinhais | KF555425 | KF421029 | KF561945 | KF420965 |
| *Nectandra megapotamica* (Spreng.) Mez | Brotto ML 504 | Clevelândia | KF555426 | KF421030 | KF561946 | KF420964 |
| *Nectandra megapotamica* (Spreng.) Mez | Brotto ML 645 | Telêmaco Borba | KF555427 | KF421031 | KF561947 |  |
| *Ocotea catharinensis* Mez | Brotto ML 663 | Quatro Barras | KF555428 | KF421032 | KF561948 | KF420963 |
| *Ocotea catharinensis* Mez | Brotto ML 664 | Quatro Barras | KF555429 | KF421033 | KF561949 |  |
| *Ocotea catharinensis* Mez | Brotto ML 694 | Diamante do Norte | KF555430 | KF421034 | KF561950 |  |
| *Ocotea odorifera* (Vell.) Rohwer | Brotto ML 284 | Morretes | KF555431 | KF421035 | KF561951 | KF420962 |
| *Ocotea odorifera* (Vell.) Rohwer | Brotto ML 628 | Jaguariaíva | KF555433 | KF421038 | KF561953 | KF420960 |
| *Ocotea odorifera* (Vell.) Rohwer | Brotto ML 620 | Ponta Grossa | KF555432 | KF421037 | KF561952 |  |
| *Ocotea odorifera* (Vell.) Rohwer | Brotto ML 414 | Curitiba |  | KF421036 |  |  |
| *Ocotea odorifera* (Vell.) Rohwer | Brotto ML 619 | Ponta Grossa |  |  |  | KF420961 |
| *Ocotea porosa* (Nees & C. Mart.) Barroso | Brotto ML 611 | Ponta Grossa |  |  |  | KF420959 |
| *Ocotea porosa* (Nees & C. Mart.) Barroso | Brotto ML 617 | Ponta Grossa | KF555435 | KF421040 | KF561954 |  |
| *Ocotea porosa* (Nees & C. Mart.) Barroso | Brotto ML 665 | Curitiba | KF555436 | KF421041 | KF561955 | KF420958 |
| *Ocotea porosa* (Nees & C. Mart.) Barroso | Brotto ML 715 | Morretes |  |  |  | KF420957 |
| *Ocotea porosa* (Nees & C. Mart.) Barroso | Brotto ML 79 | Curitiba | KF555434 | KF421039 | KF561956 | KF420956 |
| *Ocotea puberula* (Rich.) Nees | Blum CT 10-069 | Curitiba | KF555437 | KF421042 | KF561957 | KF420955 |
| *Ocotea puberula* (Rich.) Nees | Bolson M 369 | Curitiba | KF555439 | KF421044 | KF561958 | KF420953 |
| *Ocotea puberula* (Rich.) Nees | Bolson M 375 | Curitiba | KF555440 | KF421045 | KF561959 | KF420951 |
| *Ocotea puberula* (Rich.) Nees | Bolson M 327-1 | Curitiba | KF555438 | KF421043 |  | KF420954 |
| *Ocotea puberula* (Rich.) Nees | Bolson M 371-1 | Curitiba |  |  |  | KF420952 |
| *Ocotea pulchella* (Nees) Mez | Brotto ML 446 | Telêmaco Borba | KF555442 | KF421046 | KF561960 | KF420950 |
| *Ocotea pulchella* (Nees) Mez | Brotto ML 50 | Curitiba | KF555441 | KF421047 | KF561961 | KF420949 |
| *Ocotea pulchella* (Nees) Mez | Brotto ML 602 | Curitiba |  |  |  | KF420948 |
| PROTEALES |  |  |  |  |  |  |
| **Proteaceae** |  |  |  |  |  |  |
| *Roupala montana* var. *brasiliensis* (Klotzsch) K.S.Edwards | Blum CT 11-011 | Curitiba | KF555444 | KF421048 | KF561966 | KF420945 |
| MALPIGHIALES |  |  |  |  |  |  |
| **Erytroxylaceae** |  |  |  |  |  |  |
| *Erythroxylum deciduum* A. St. | Blum CT 11-047 | Tijucas do Sul |  | KF421049 | KF561912 | KF421001 |
| *Erythroxylum deciduum* A. St. | Bolson M 399 | Ponta Grossa |  | KF421050 | KF561913 | KF421000 |
| *Erythroxylum deciduum* A. St. | Bolson M 421 | Tibagi |  |  |  | KF420999 |
| OXALIDALES |  |  |  |  |  |  |
| **Cunoniaceae** |  |  |  |  |  |  |
| *Lamanonia ternata* Vell. | Blum CT 10-127 | Quatro Barras | KF555403 | KF421051 | KF561922 | KF420994 |
| *Lamanonia ternata* Vell. | Bolson M 456 | Honório Serpa | KF555404 | KF421055 | KF561923 | KF420991 |
| *Lamanonia ternata* Vell. | Blum CT 10-128 | Quatro Barras |  | KF421052 |  | KF420993 |
| *Lamanonia ternata* Vell. | Blum CT 10-141 | Curitiba |  | KF421054 |  | KF420992 |
| *Lamanonia ternata* Vell. | Bolson M 462 | Piraquara |  | KF421056 |  | KF420990 |
| *Lamanonia ternata* Vell. | Blum CT 11-022 | Tijucas do Sul |  | KF421053 |  |  |
| FABALES |  |  |  |  |  |  |
| **Fabaceae** |  |  |  |  |  |  |
| *Inga marginata* Willd. | Blum CT 11-016 | Curitiba | KF555397 | KF421057 | KF561916 |  |
| *Mimosa scabrella* Benth. | Blum CT 10-045 | Campo Largo | KF555411 | KF421058 | KF561930 | KF420983 |
| *Mimosa scabrella* Benth. | Bolson M 353 | Curitiba | KF555415 | KF421063 | KF561932 | KF420980 |
| *Mimosa scabrella* Benth. | Bolson M 347 | Curitiba | KF555414 | KF421062 | KF561931 |  |
| *Mimosa scabrella* Benth. | Blum CT 10-046 | Campo Largo | KF555412 | KF421059 |  | KF420982 |
| *Mimosa scabrella* Benth. | Blum CT 10-047 | Campo Largo | KF555413 | KF421060 |  | KF420981 |
| *Mimosa scabrella* Benth. | Blum CT 10-049 | Campo Largo |  | KF421061 |  |  |
| ROSALES |  |  |  |  |  |  |
| **Rosaceae** |  |  |  |  |  |  |
| *Prunus myrtifolia* (L.) Urb. | Bolson M 360 | Curitiba |  | KF421064 | KF561964 | KF420947 |
| *Prunus myrtifolia* (L.) Urb. | Bolson M 368 | Curitiba | KF555443 | KF421065 |  | KF420946 |
| *Prunus myrtifolia* (L.) Urb. | Bolson M 393 | Curitiba |  | KF421067 | KF561965 |  |
| *Prunus myrtifolia* (L.) Urb. | Bolson M 375-1 | Curitiba |  | KF421066 |  |  |
| MYRTALES |  |  |  |  |  |  |
| **Lythraceae** |  |  |  |  |  |  |
| *Lafoensia pacari* A. St. | Blum CT 11-017 | Curitiba | KF555401 | KF421068 | KF561920 |  |
| *Lafoensia pacari* A. St. | Lima DF 117 | Curitiba | KF555402 | KF421069 | KF561921 | KF420995 |
| **Myrtaceae** |  |  |  |  |  |  |
| *Campomanesia xanthocarpa* O. Berg | Blum CT 10-101 | Curitiba | KF555386 | KF421070 | KF561905 | KF421010 |
| *Campomanesia xanthocarpa* O. Berg | Blum CT 10-109 | Colombo | KF555387 | KF421071 | KF561906 | KF421009 |
| *Campomanesia xanthocarpa* O. Berg | Blum CT 10-100 | Curitiba |  |  | KF561904 | KF421011 |
| *Myrcia splendens* (Sw.) DC. | Blum CT 11-024 | Tijucas do Sul | KF555416 | KF421074 | KF561935 | KF420977 |
| *Myrcia splendens* (Sw.) DC. | Blum CT 11-003 | Piraquara |  | KF421073 | KF561934 | KF420978 |
| *Myrcia splendens* (Sw.) DC. | Blum CT 10-098 | Curitiba |  | KF421072 | KF561933 | KF420979 |
| SAPINDALES |  |  |  |  |  |  |
| **Anacardiaceae** |  |  |  |  |  |  |
| *Lithrea molleoides* (Vell.) Engl. | Bolson M 404 | Ponta Grossa | KF555405 | KF421075 | KF561924 | KF420989 |
| *Schinus terebinthifolius* Raddi | Bolson M 328-1 | Curitiba | KF555445 | KF421077 | KF561967 | KF420943 |
| *Schinus terebinthifolius* Raddi | Bolson M 332-1 | Campina Grade do Sul | KF555447 | KF421079 | KF561968 | KF420940 |
| *Schinus terebinthifolius* Raddi | Bolson M 331-1 | Curitiba | KF555446 | KF421078 |  | KF420942 |
| *Schinus terebinthifolius* Raddi | Blum CT 11-013 | Curitiba |  | KF421076 |  | KF420944 |
| *Schinus terebinthifolius* Raddi | Bolson M 331-2 | Curitiba |  |  |  | KF420941 |
| *Schinus terebinthifolius* Raddi | Bolson M 332-2 | Curitiba |  |  |  | KF420939 |
| **Meliaceae** |  |  |  |  |  |  |
| *Cabralea canjerana* (Vell.) Mart. | Blum CT 10-102 | Curitiba | KF555384 | KF421080 | KF561902 | KF421013 |
| *Cabralea canjerana* (Vell.) Mart. | Bolson M 460 | Ponta Grossa | KF555385 | KF421081 | KF561903 | KF421012 |
| *Cedrela fissilis* Vell. | Bolson M 487 | Curitiba | KF555388 | KF421082 | KF561907 | KF421008 |
| **Rutaceae** |  |  |  |  |  |  |
| *Zanthoxylum rhoifolium* Lam. | Blum CT 10-106 | Colombo | KF555448 | KF421083 | KF561971 | KF420934 |
| **Sapindaceae** |  |  |  |  |  |  |
| *Cupania vernalis* Cambess. | Blum CT 11-040 | Tijucas do Sul | KF555391 | KF421086 | KF561910 | KF421004 |
| *Cupania vernalis* Cambess. | Bolson M 340-1 | Curitiba | KF555392 | KF421087 | KF561911 | KF421003 |
| *Cupania vernalis* Cambess. | Brotto ML 530 | Clevelândia | KF555393 | KF421088 |  | KF421002 |
| *Cupania vernalis* Cambess. | Blum CT 11-038 | Tijucas do Sul | KF555390 | KF421084 |  |  |
| *Cupania vernalis* Cambess. | Blum CT 11-039 | Tijucas do Sul |  | KF421085 |  | KF421005 |
| *Matayba elaeagnoides* Radlk. | Blum CT 10-107 | Colombo | KF555408 | KF421089 | KF561927 | KF420986 |
| *Matayba elaeagnoides* Radlk. | Blum CT 10-132 | Campo Largo | KF555409 | KF421090 | KF561928 | KF420985 |
| *Matayba elaeagnoides* Radlk. | Bolson M 345 | Curitiba | KF555410 | KF421091 | KF561929 | KF420984 |
| MALVALES |  |  |  |  |  |  |
| **Malvaceae** |  |  |  |  |  |  |
| *Luehea divaricata* Mart. | Bolson M 349 | Curitiba | KF555406 | KF421092 | KF561925 | KF420988 |
| *Luehea divaricata* Mart. | Bolson M 471 | Curitiba | KF555407 | KF421093 | KF561926 | KF420987 |
| ERICALES |  |  |  |  |  |  |
| **Myrsinaceae** |  |  |  |  |  |  |
| *Myrsine umbellata* Mart. | Blum CT 10-024 | Tijucas do Sul | KF555417 | KF421094 | KF561936 | KF420976 |
| *Myrsine umbellata* Mart. | Blum CT 10-076 | Tijucas do Sul | KF555418 | KF421096 | KF561938 |  |
| *Myrsine umbellata* Mart. | Blum CT 10-072 | Curitiba |  | KF421095 | KF561937 | KF420975 |
| *Myrsine umbellata* Mart. | Bolson M 401 | Ponta Grossa | KF555419 |  |  | KF420974 |
| LAMIALES |  |  |  |  |  |  |
| **Bignoniaceae** |  |  |  |  |  |  |
| *Jacaranda puberula* Cham. | Blum CT 339-1 | Curitiba | KF555399 | KF421098 | KF561918 | KF420997 |
| *Jacaranda puberula* Cham. | Blum CT 339-2 | Curitiba | KF555400 | KF421099 | KF561919 | KF420996 |
| *Jacaranda puberula* Cham. | Blum CT 10-104 | Colombo | KF555398 | KF421097 | KF561917 |  |
| **Lamiaceae** |  |  |  |  |  |  |
| *Vitex megapotamica* (Spreng.) Moldenke | Blum CT 10-136 | Curitiba |  | KF421100 | KF561969 | KF420938 |
| *Vitex megapotamica* (Spreng.) Moldenke | Bolson M 321 | Curitiba |  | KF421104 | KF561970 |  |
| *Vitex megapotamica* (Spreng.) Moldenke | Blum CT 10-137 | Curitiba |  | KF421101 |  | KF420937 |
| *Vitex megapotamica* (Spreng.) Moldenke | Blum CT 10-140 | Curitiba |  | KF421102 |  | KF420936 |
| *Vitex megapotamica* (Spreng.) Moldenke | Bolson M 326 | Curitiba |  | KF421105 |  | KF420935 |
| *Vitex megapotamica* (Spreng.) Moldenke | Blum CT 11-023 | Tijucas do Sul |  | KF421103 |  |  |
| AQUIFOLIALES |  |  |  |  |  |  |
| **Aquifoliaceae** |  |  |  |  |  |  |
| *Ilex* *theezans* Mart. | Blum CT 11-044 | Tijucas do Sul | KF555396 | KF421108 | KF561915 | KF420998 |
| *Ilex* *theezans* Mart. | Blum CT 10-125 | Quatro Barras | KF555395 | KF421107 | KF561914 |  |
| *Ilex* *theezans* Mart. | Blum CT 10-086 | Tijucas do Sul | KF555394 | KF421106 |  |  |
