# Supplementary material for: ITS and trnH-psbA as Efficient DNA Barcodes to Identify Threatened Commercial Woody Angiosperms from Southern Brazilian Atlantic Rainforests
Source: PLoS One. 2015 Dec 2;10(12):e0143049. doi: 10.1371/journal.pone.0143049 (PMC4704546; doi:10.1371/journal.pone.0143049)
Supplement: S2 Table — The complete references can be found in the text. (DOCX) [file pone.0143049.s003.docx]

**S2 Table. Primers and PCR conditions for plastid and nuclear DNA sequence amplifications in this study.** The complete references can be found in the text.

| **Region** | **Primers** | **Sequence (5’-3’)** | **PCR Conditions** | **Reference** |
| --- | --- | --- | --- | --- |
| *matK* | *3F_KIM_ f* | 5’- CGT ACA GTA CTT TTG TGT TTA CGA G- 3’ | 94°C 1min  (94°C 30seg 53°C 40seg 72°C 40seg) x40  72°C 5min | Ki-Joong Kim, http://www.barcoding.si.edu/plant_working_group.html |
|  | *1R_KIM_ r* | 5’- ACC CAG TCC ATC TGG AAA TCT TGG TTC- 3’ |  | Ki-Joong Kim, http://www.barcoding.si.edu/plant_working_group.html |
|  | *2.1F* | 5’- CCT ATC CAT CTG GAA ATC TTA G- 3’ |  | Ford *et al.* (2009) |
|  | *5R* | 5’- GTT CTA GCA CAA GAA AGT CG- 3’ |  | Ford *et al.* (2009) |
| *rbcL* | *rbcLa_f* | 5’- ATG TCA CCA CAA ACA GAG ACT AAA GC- 3’ | 94°C 1min  (94°C 30seg 53°C 40seg 72°C 40seg) x40  72°C 5min | Levin *et al.* (2003) |
|  | *rbcLa_r* | 5’-GTA AAA TCA AGT CCA CCR CG- 3’ |  | Kress & Erickson (2007) |
|  |  |  |  |  |
| *psbA- trnH* | *psbA3_f* | 5’- GTT ATG CAT GAA CGT AAT GCT C- 3’ | 95°C 3min  (95°C 30seg 55°C 1min 72°C 1,5min) x35  72°C 4min | Sang *et al.* (1997) |
|  | *trnHf_05* | 5’- CGC GCA TGG TGG ATT CAC AAT CC- 3’ |  | Tate & Simpson (2003) |
| ITS | ITS75 | 5’- TAT GCT TAA ACT CCA CGG G- 3’ | 94°C 1min  (94°C 30seg 51°C 40seg 72°C 40seg) x40  72°C 5min | Desfeaux *et al.* (1996) |
|  | ITS92 | 5’- AAG GTT TCC GTA GGT GAA- 3’ |  | Desfeaux *et al.* (1996) |
|  | ITS18_F | 5’- GTC CAC TGA ACC TTA TCA TTT AGA GG- 3’ |  | Beyra-Matos & Lavin (1999) |
|  | ITS26_R | 5’-GCC GTT ACT AAG GGA ATC CTT GTT AG- 3’ |  | Käss & Wink (1997) |
