# Supplementary material for: ITS and trnH-psbA as Efficient DNA Barcodes to Identify Threatened Commercial Woody Angiosperms from Southern Brazilian Atlantic Rainforests
Source: PLoS One. 2015 Dec 2;10(12):e0143049. doi: 10.1371/journal.pone.0143049 (PMC4704546; doi:10.1371/journal.pone.0143049)
Supplement: S3 Table — (DOCX) [file pone.0143049.s004.docx]

**S3 Table. Percentage of species-specific clusters (SSC) and non-species-specific clusters (NSSC) using different tree-based methods (NJ, MP, and ML) with different bootstrap support for single barcode regions and different combinations.**

| **Barcode regions** | **SSC (%)** | | | **NSSC (%)** |
| --- | --- | --- | --- | --- |
|  | **<70** | **70 ≤99.9** | **=100** |  |
| NJ |  |  |  |  |
| *matK* | 10.0 | 15.0 | 35.0 | 40.0 |
| *trnH-psbA* | 4.0 | 48.0 | 8.0 | 40.0 |
| *rbcL* | 4.0 | 32.0 | 36.0 | 28.0 |
| ITS | 4.5 | 36.3 | 40.9 | 18.2 |
| *matK + rbcL* | 0.0 | 25.0 | 40.0 | 35.0 |
| *trnH-psbA +* ITS | 4.5 | 9.0 | 68.2 | 18.2 |
| *matK + trnH-psbA + rbcL* | 4.0 | 4.0 | 60.0 | 32.0 |
| *matK + trnH-psbA + rbcL +* ITS | 4.2 | 4.2 | 58.3 | 33.3 |
| ML |  |  |  |  |
| *matK* | 5.0 | 10.0 | 40.0 | 45.0 |
| *trnH-psbA* | 0.0 | 12.0 | 52.0 | 36.0 |
| *rbcL* | 0.0 | 16.0 | 56.0 | 28.0 |
| ITS | 4.5 | 40.8 | 50.0 | 4.5 |
| *matK + rbcL* | 0.0 | 10.0 | 55.0 | 35.0 |
| *trnH-psbA +* ITS | 0.0 | 22.6 | 68.2 | 9.1 |
| *matK + trnH-psbA + rbcL* | 0.0 | 12.0 | 64.0 | 24.0 |
| *matK + trnH-psbA + rbcL +* ITS | 8.3 | 0.0 | 75.0 | 16.7 |
| MP |  |  |  |  |
| *matK* | 0.0 | 10.0 | 35.0 | 55.0 |
| *trnH-psbA* | 0.0 | 12.0 | 60.0 | 28.0 |
| *rbcL* | 4.0 | 52.0 | 16.0 | 28.0 |
| ITS | 0.0 | 50.0 | 45.5 | 4.5 |
| *matK + rbcL* | 0.0 | 10.0 | 55.0 | 35.0 |
| *trnH-psbA +* ITS | 9.1 | 27.2 | 59.1 | 4.5 |
| *matK + trnH-psbA + rbcL* | 0.0 | 32.0 | 44.0 | 24.0 |
| *matK + trnH-psbA + rbcL +* ITS | 0.0 | 8.3 | 66.7 | 25.0 |
